# Requirements for the robust operant conditioning of neural firing rates

**DOI:** 10.1186/1471-2202-14-S1-P48

**Published:** 2013-07-08

**Authors:** Robert R Kerr, David B Grayden, Doreen A Thomas, Matthieu Gilson, Anthony N Burkitt

**Affiliations:** 1 NeuroEngineering Laboratory, Dept. of Electrical & Electronic Engineering, University of Melbourne, Australia; 2 Centre for Neural Engineering, University of Melbourne, Australia; 3 NICTA, Victoria Research Lab, University of Melbourne, Melbourne, Australia; 4 Bionics Institute, Melbourne, Australia; 5 Department of Mechanical Engineering, University of Melbourne, Melbourne, Australia; 6 Laboratory for Neural Circuit Theory, RIKEN Brain Science Institute, Saitama, Japan

## 

**Figure 1:**
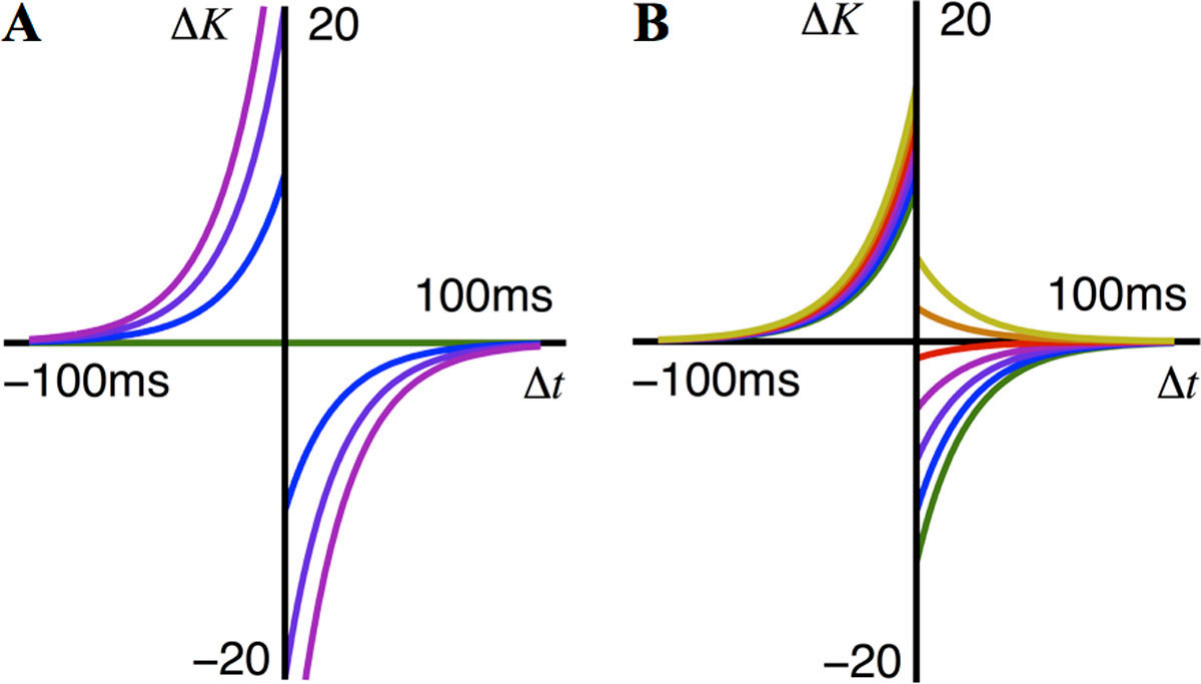
**Effective STDP learning windows that show the synaptic change, *ΔK*, caused by a spike pair with the timing difference, *Δt*, between the pre- and post-synaptic spikes**. The different windows are for different reward signal levels (y = 0, 1, 2, 3, 4, 5, and 6, shown in green, blue, purple, magenta, red, orange, and yellow lines, respectively). **A: **Classical RSTDP has no LTD or LTP when there is no reward and increasing amounts of both as reward increases. **B: **Generalized RSTDP allows separate modulation of LTP and LTD and non-zero LTP and LTD when there is no reward. Shown here is generalized RSTDP parameterized to match the effect dopamine has been observed to have on STDP [4]. Switching of LTD to LTP for high reward values is exhibited.

Operant conditioning experiments have shown that changes in the firing rates of individual neurons in the motor cortex of monkeys can be elicited [1,2]. In these experiments, the firing rate of the neurons were measured using an implanted electrode, and the monkeys were presented with feedback based on these rates and rewarded for increasing them. Behavioral learning such as this is assumed to be due to plasticity at the synaptic level and reward-modulated spike-timing-dependent plasticity (RSTDP) has previously been proposed as such a model [3]. In this study, we propose a generalization of the existing RSTDP model (classical RSTDP) that can account for experiments where dopamine differentially modulates the amplitude of long-term potentiation and depression (LTP and LTD) [4]. Using analytical techniques and numerical simulations with leaky integrate-and-fire (LIF) neurons, we compare the classical RSTDP (see Figure 1A) with our generalized model (see Figure 1B). We consider the potential for these models to elicit the increased firing rates observed in operant conditioning experiments [1,2] and find two requirements. The first requirement is that, relative to their base level amplitudes, the strengthening of LTP by the reward signal must be greater than the strengthening of LTD. Classical RSTDP cannot exhibit this and, contrary to previous studies [3], we predict that it consequently cannot robustly elicit an increased firing rate. The second requirement is that the reinforced neuron must be able to exhibit short inter-spike intervals (ISIs) relative to its mean ISI. For the LIF neurons we consider, this corresponds to being in a fluctuation-driven regime, such as receiving a balance of excitatory and inhibitory inputs. The findings of this study are consistent with existing experimental studies and they also make testable predictions for possible future experiments.
